# Resection of Ribs in Apical Phthisis

**Published:** 1898-11-26

**Authors:** 


### RESECTION OF RIBS IN APICAL PHTHISIS.

Notwithstanding the fatality and destructive nature of pulmonary phthisis as ordinarily met with, the clinical history of individual cases shows what post-mortem experience proves with still greater exactness, that the course of the disease is an intermittent one, its various steps and stages being interrupted by repeated efforts at recovery; and, indeed, that even while the disease is actually spreading into new portions of the lung, the parts already affected may be contracting and doing their best to close up and to heal. In dealing, then, with a disease which shows so strong a tendency towards recovery, and sometimes appears to make such prodigious efforts to cure itself, it is important to discover, if possible, why it so often falls just short of doing so. This is a question to the solution of which Mr. Norman Porritt[1] applies himself with much care. Why does the lesion, surrounded as it is with an abundant supply of nutritive material, not heal? I venture to suggest, he

[1] Lancet, Nov. 19.

says, that the reason is a mechanical one. The damaged and diseased lung shrinks as far as it can, drawing in the ribs and distorting the chest in the manner which is familiar. If the lung were surrounded with a wholly fleshy case instead of a rigid bony framework, we should have many more examples of healed phthisical lesions to add to those found in the post-mortem rooms. The lung becomes adherent to the chest walls, and the unyielding ribs hold the walls of the cavity apart. Nature does her best, the lung contracts as far as it can, and the disease becomes for the time arrested. Some such cases, in fact, would heal but for the ribs, and these would seem to be cases in which we might well return the lead which nature gives us, and endeavour to place the lung in such circumstances that the healing process may be carried to the fullest possible extent. Mr. Porritt therefore proposes that portions of the ribs should be removed both back and front from the upper part of the chest. Two soft yielding surfaces are thus formed with a loose length of rib between, the area from which the portions of rib are taken being left free to follow the contracting and healing lung, while the floating pieces of rib can be drawn in bodily towards the spine or in any other direction that the shrinking lung takes. Three cases are related in which Mr. Porritt has done this operation.

We cannot say that so far as these particular cases go there is much that is encouraging. Nevertheless, it is quite possible that in certain stiff-ribbed individuals some such proceeding may turn out to be of use. Where, however, the chest walls are fairly flexible, as in young people, we fancy that they will generally be found to yield sufficiently to allow of the complete cicatrisation in moderate degrees of tuberculous infiltration, and that in such cases the real difficulty will not lie in the ribs. On the other hand, where excavation has gone to such an extent that the chest walls cannot fall in sufficiently to allow the cavities to close, it will frequently be found that the disease extends far beyond the upper portions of the lungs. Still, here and there cases may be met with in which it may be as advantageous to remove rib to allow of the closing in of a tuberculous cavity as it is to do the same thing for the sake of promoting the closure of an empyæma with rigid walls. Such cases, however, cannot, we fancy, be very common.

